# AI in Medical Imaging Informatics: Current Challenges and Future Directions

**DOI:** 10.1109/JBHI.2020.2991043

**Published:** 2020-07

**Authors:** Andreas S. Panayides, Amir Amini, Nenad D. Filipovic, Ashish Sharma, Sotirios A. Tsaftaris, Alistair Young, David Foran, Nhan Do, Spyretta Golemati, Tahsin Kurc, Kun Huang, Konstantina S. Nikita, Ben P. Veasey, Michalis Zervakis, Joel H. Saltz, Constantinos S. Pattichis

**Affiliations:** Department of Computer Science, University of Cyprus, 1678 Nicosia, Cyprus; Electrical and Computer Engineering Department, University of Louisville, Louisville, KY 40292 USA; University of Kragujevac, 2W94+H5 Kragujevac, Serbia; Emory University Atlanta, GA 30322 USA; School of Engineering, The University of Edinburgh, EH9 3FG, U.K.; The Alan Turing Institute, U.K.; Department of Anatomy and Medical Imaging, University of Auckland, Auckland 1142, New Zealand; Department of Pathology and Laboratory Medicine, Robert Wood Johnson Medical School, Rutgers, The State University of New Jersey, Piscataway, NJ 08854 USA; U.S. Department of Veterans Affairs Boston Healthcare System, Boston, MA 02130 USA; Medical School, National and Kapodistrian University of Athens, Athens 10675, Greece; Stony Brook University,, Stony Brook, NY 11794 USA; School of Medicine, Regenstrief Institute, Indiana University, IN 46202 USA; Biomedical Simulations and Imaging Lab, School of Electrical and Computer Engineering, National Technical University of Athens, Athens 157 80, Greece; Electrical and Computer Engineering Department, University of Louisville, Louisville, KY 40292 USA; Technical University of Crete, Chania 73100, Crete, Greece; Stony Brook University,, Stony Brook, NY 11794 USA; Department of Computer Science of the University of Cyprus, 1678 Nicosia, Cyprus, and also with the Research Centre on Interactive Media, Smart Systems and Emerging Technologies (RISE CoE), 1066 Nicosia, Cyprus

**Keywords:** Medical Imaging, Image Analysis, Image Classification, Image Processing, Image Segmentation, Image Visualization, Integrative Analytics, Machine Learning, Deep Learning, Big Data

## Abstract

This paper reviews state-of-the-art research solutions across the spectrum of medical imaging informatics, discusses clinical translation, and provides future directions for advancing clinical practice. More specifically, it summarizes advances in medical imaging acquisition technologies for different modalities, highlighting the necessity for efficient medical data management strategies in the context of AI in big healthcare data analytics. It then provides a synopsis of contemporary and emerging algorithmic methods for disease classification and organ/ tissue segmentation, focusing on AI and deep learning architectures that have already become the de facto approach. The clinical benefits of in-silico modelling advances linked with evolving 3D reconstruction and visualization applications are further documented. Concluding, integrative analytics approaches driven by associate research branches highlighted in this study promise to revolutionize imaging informatics as known today across the healthcare continuum for both radiology and digital pathology applications. The latter, is projected to enable informed, more accurate diagnosis, timely prognosis, and effective treatment planning, underpinning precision medicine.

## I. Introduction

MEDICAL imaging informatics covers the application of information and communication technologies (ICT) to medical imaging for the provision of healthcare services. A wide-spectrum of multi-disciplinary medical imaging services have evolved over the past 30 years ranging from routine clinical practice to advanced human physiology and pathophysiology. Originally, it was defined by the Society for Imaging Informatics in Medicine (SIIM) as follows [1]–[3]:

“Imaging informatics touches every aspect of the imaging chain from image creation and acquisition, to image distribution and management, to image storage and retrieval, to image processing, analysis and understanding, to image visualization and data navigation; to image interpretation, reporting, and communications. The field serves as the integrative catalyst for these processes and forms a bridge with imaging and other medical disciplines.”

The objective of medical imaging informatics is thus, according to SIIM, to improve efficiency, accuracy, and reliability of services within the medical enterprise [3], concerning medical image usage and exchange throughout complex healthcare systems [4]. In that context, linked with the associate technological advances in big-data imaging, -omics and electronic health records (EHR) analytics, dynamic workflow optimization, context-awareness, and visualization, a new era is emerging for medical imaging informatics, prescribing the way towards precision medicine [5]–[7]. This paper provides an overview of prevailing concepts, highlights challenges and opportunities, and discusses future trends.

Following the key areas of medical imaging informatics in the definition given above, the rest of the paper is organized as follows: Section II covers advances in medical image acquisition highlighting primary imaging modalities used in clinical practice. Section III discusses emerging trends pertaining to the data management and sharing in the medical imaging big data era. Then, Section IV introduces emerging data processing paradigms in radiology, providing a snapshot of the timeline that has today led to increasingly adopting AI and deep learning analytics approaches. Likewise, Section V reviews the state-of-the-art in digital pathology. Section VI describes the challenges pertaining to 3D reconstruction and visualization in view of different application scenarios. Digital pathology visualization challenges are further documented in this section, while in-silico modelling advances are presented next, debating the need of introducing new integrative, multi-compartment modelling approaches. Section VII discusses the need of integrative analytics and discusses emerging radiogenomics paradigm for both radiology and digital pathology approaches. Finally, Section VIII provides the concluding remarks along with a summary of future directions.

## II. Image Formation and Acquisition

**Fig. 1.**
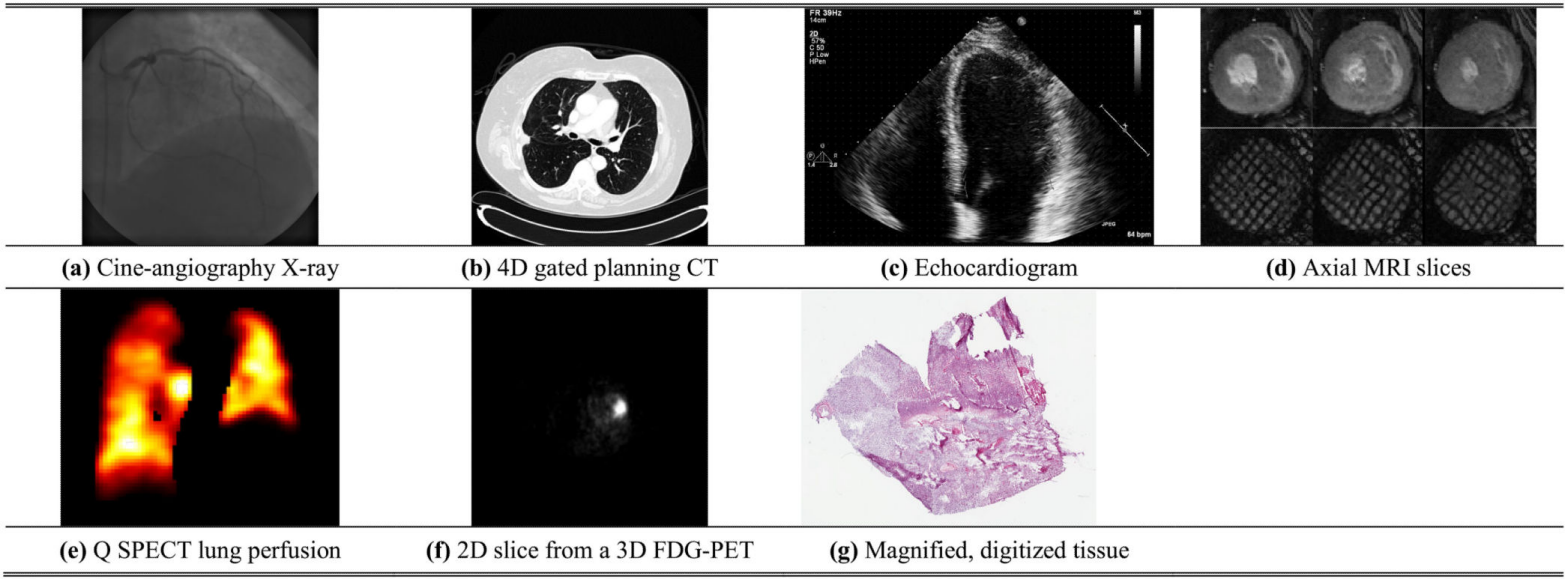
Typical medical imaging examples. (a) Cine angiography X-ray image after injection of iodinated contrast; (b) An axial slice of a 4D, gated planning CT image taken before radiation therapy for lung cancer; (c) Echocardiogram – 4 chamber view showing the 4 ventricular chambers (ventricular apex located at the top); (d) First row – axial MRI slices in diastole (left), mid-systole (middle), and peak systolic (right). Note the excellent contrast between blood pool and left ventricular myocardium. Second row –tissues tagged MRI slices at the same slice location and time point during the cardiac cycle. The modality creates noninvasive magnetic markers within the moving tissue [40]; (e) A typical Q SPECT image displaying lung perfusion in a lung-cancer patient; (f) A 2D slice from a 3D FDG-PET scan that shows a region of high glucose activity corresponding to a thoracic malignancy; (g) A magnified, digitized image of brain tissue to look for signs of Glioblastoma (taken from TCGA Glioblastoma Multiforme collection (https://cancergenome.nih.gov/).

Biomedical imaging has revolutionized the practice of medicine with unprecedented ability to diagnose disease through imaging the human body and high-resolution viewing of cells and pathological specimens. Broadly speaking, images are formed through interaction of electromagnetic waves at various wavelengths (energies) with biological tissues for modalities other than Ultrasound, which involves use of mechanical sound waves. Images formed with high-energy radiation at shorter wavelength such as X-ray and Gamma-rays at one end of the spectrum are ionizing whereas at longer wavelength - optical and still longer wavelength - MRI and Ultrasound are nonionizing. The imaging modalities covered in this section are X-ray, ultrasound, magnetic resonance (MR), X-ray computed tomography (CT), nuclear medicine, and high-resolution microscopy [8], [9] (see Table I). Fig. 1 shows some examples of images produced by these modalities.

X-ray imaging’s low cost and quick acquisition time has led to it being one of the most commonly used imaging techniques. The image is produced by passing X-rays generated by an X-ray source through the body and detecting the attenuated X-rays on the other side via a detector array; the resulting image is a 2D projection with resolutions down to 100 microns and where the intensities are indicative of the degree of X-ray attenuation [9]. To improve visibility, iodinated contrast agents that attenuate X-rays are often injected into a region of interest (e.g., imaging arterial disease through fluoroscopy). Phase-contrast X-ray imaging can also improve soft-tissue image contrast by using the phase-shifts of the X-rays as they traverse through the tissue [10]. X-ray projection imaging has been pervasive in cardiovascular, mammography, musculoskeletal, and abdominal imaging applications among others [11].

Ultrasound imaging (US) employs pulses in the range of 1–10 MHz to image tissue in a noninvasive and relatively inexpensive way. The backscattering effect of the acoustic pulse interacting with internal structures is used to measure the echo to produce the image. Ultrasound imaging is fast, enabling, for example, real-time imaging of blood flow in arteries through the Doppler shift. A major benefit of ultrasonic imaging is that no ionizing radiation is used, hence less harmful to the patient. However, bone and air hinder the propagation of sound waves and can cause artifacts. Still, ultrasound remains one of the most used imaging techniques employed extensively for real-time cardiac and fetal imaging [11]. Contrast-enhanced ultrasound has allowed for greater contrast and imaging accuracy with the use of injected microbubbles to increase reflection in specific areas in some applications [12]. Ultrasound elasticity imaging has also been used for measuring the stiffness of tissue for virtual palpation [13]. Importantly, ultrasound is not limited to 2D imaging and use of 3D and 4D imaging is expanding, though with reduced temporal resolution [14].

MR imaging [15] produces high spatial resolution volumetric images primarily of Hydrogen nuclei, using an externally applied magnetic field in conjunction with radio-frequency (RF) pulses which are non-ionizing [1]. MRI is commonly used in numerous applications including musculoskeletal, cardiovascular, and neurological imaging with superb soft-tissue contrast [16], [17]. Additionally, functional MRI has evolved into a large sub-field of study with applications in areas such as mapping the functional connectivity in the brain [18]. Similarly, diffusion-weighted MRI images the diffusion of water molecules in the body and has found much use in neuroimaging and oncology applications [19]. Moreover, Magnetic Resonance Elastography (MRE) allows virtual palpation with significant applications in liver fibrosis [20], while 4D flow methods permit exquisite visualization of flow in 3*D* + *t* [17], [21]. Techniques that accelerate the acquisition time of scans, e.g. compressed sensing, non-Cartesian acquisitions [22], and parallel imaging [23], have led to increased growth and utilization of MR imaging. In 2017, 36 million MRI scans were performed in the US alone [24].

X-ray CT imaging [25] also offers volumetric scans like MRI. However, CT CT produces a 3D image via the construction of a set of 2D axial slices of the body. Similar to MRI, 4D scans are also possible by gating to the ECG and respiration. Improved solid-state detectors, common in modern CT scanners, have improved spatial resolutions to 0.25 mm [26], while multiple detector rows enable larger spatial coverage with slice thicknesses down to 0.625 mm. Spectral computed tomography (SCT) utilizes multiple X-ray energy bands that are used to produce distinct attenuation data sets of the same organs. The resulting data permit material composition analysis for a more accurate diagnosis of disease [27]. CT is heavily used due to its quick scan time and excellent resolution, in spite concerns of radiation dosage. Around 74 million CT studies were performed in the US alone in 2017 [24], and this number is bound to grow due to CT’s increased applications in screening in emergency care.

In contrast to transmission energy used in X-ray based modalities, nuclear medicine is based on imaging gamma rays that are emitted through radioactive decay of radioisotopes introduced in the body. The radioisotopes emit radiation that is detected by an external camera before being reconstructed into an image [11]. Single photon emission computed tomography (SPECT) and positron emission tomography (PET) are common techniques in nuclear medicine. Both produce 2D image slices that can be combined into a 3D volume; however, PET imaging uses positron-emitting radiopharmaceuticals that produce two gamma rays when a released positron meets a free electron. This allows PET to produce images with higher signal-to-noise ratio and spatial resolution as compared to SPECT [9]. PET is commonly used in combination with CT imaging (PET/CT) [28] and more recently PET/MR [29] to provide complementary information of a potential abnormality. The use of fluorodeoxyglucose (FDG) in PET has led to a powerful method for diagnosis and cancer staging. Time-of-flight PET scanners offer improved image quality and higher sensitivity during shorter scan times over conventional PET and are particularly effective for patients with a large body habitus [30].

Last but not least, the use of microscopy in imaging of cells and tissue sections is of paramount importance for disease diagnosis, e.g. for biopsy and/ or surgical specimens. Conventional tissue slides contain one case per slide. A single tissue specimen taken from a patient is fixated on a glass slide and stained. Staining enhances visual representation of tissue morphology, enabling a pathologist to view and interpret the morphology more accurately. Conventional staining methods include Hematoxylin and Eosin (H&E), which is the most common staining system and stains nuclei, and immunohistochemical staining systems. Light microscopes use the combination of an illuminator and two or more lenses to magnify samples up to 1,000x although lower magnifications are often used in histopathology. This allows objects to be viewed at resolutions of approximately 0.2 *μ*m and acts as the primary tool in diagnosing histopathology. Light microscopy is often used to analyze biopsy samples for potential cancers as well as for studying tissue-healing processes [1], [31].

While conventional microscopy uses the principle of transmission to view objects, the emission of light at a different wavelength can help increase contrast in objects that fluoresce by filtering out the excitatory light and only viewing the emitted light – called fluorescence microscopy [32]. Two-photon fluorescence imaging uses two photons of similar frequencies to excite molecules which allows for deeper penetration of tissue and lower phototoxicity (damage to living tissue caused by the excitation source) [33]. These technologies have seen use in neuro [34], [33] and cancer [35] imaging among other areas.

Another tissue slide mechanism is the Tissue Microarray (TMA). TMA technology enables investigators to extract small cylinders of tissue from histological sections and arrange them in a matrix configuration on a recipient paraffin block such that hundreds can be analyzed simultaneously [36]. Each spot on a tissue microarray is a complex, heterogeneous tissue sample, which is often prepared with multiple stains. While single case tissue slides remain the most common slide type, TMA is now recognized as a powerful tool, which can provide insight regarding the underlying mechanisms of disease progression and patient response to therapy. With recent advances in immuneoncology, TMA technology is rapidly becoming indispensable and augmenting single case slide approaches. TMAs can be imaged using the same whole slide scanning technologies used to capture images of single case slides. Whole slide scanners are becoming increasingly ubiquitous in both research and remote pathology interpretation settings [37].

For *in-vivo* imaging, Optical Coherence Tomography (OCT) can produce 3D images from a series of cross-sectional optical images by measuring the echo delay time and intensity of backscattered light from internal microstructures of the tissue in question [38]. Hyperspectral imaging is also used by generating an image based on several spectra (sometimes hundreds) of light to gain a better understanding of the reflectance properties of the object being imaged [39].

The challenges and opportunities in the area of biomedical imaging include continuing acquisitions at faster speeds and lower radiation dose in the case of anatomical imaging methods. Variations in imaging parameters (e.g. in-plane resolution, slice thickness, etc.) – which were not discussed – may have strong impacts on image analysis and should be considered during algorithm development. Moreover, the prodigious amount of imaging data generated causes a significant need for informatics in the storage and transmission as well as in the analysis and automated interpretation of the data, underpinning the use of big data science in improved utilization and diagnosis.

## III. Interoperable and Fair Data Repositories For Reproducible, Extensible and Explainable Research

Harnessing the full potential of available big data for healthcare innovation necessitates a change management strategy across both research institutions and clinical sites. In its present form, heterogeneous healthcare data ranging from imaging, to genomic, to clinical data, that are further augmented by environmental data, physiological signals and other, cannot be used for integrative analysis (see Section VII) and new hypothesis testing. The latter is attributed to a number of factors, a non-exhaustive list extending to the data being scattered across and within institutions in a poorly indexed fashion, not being openly-available to the research community, and not being well-curated nor semantically annotated. Additionally, these data are typically semi- or un- structured, adding a significant computational burden for constituting them data mining ready.

A cornerstone for overcoming the aforementioned limitations relies on the establishment of efficient, enterprise-wide clinical data repositories (CDR). CDRs can systematically aggregate information arising from: (i) Electronic Health and Medical Records (EHR/ EMR; term used interchangeably); (ii) Radiology and Pathology archives (relying on picture archive and communication systems (PACS)), (iii) a wide range of genomic sequencing devices, Tumor Registries, and Biospecimen Repositories, as well as (iv) Clinical Trial Management Systems (CTMS). Here, it is important to note that EHR/ EMR are now increasingly used as the umbrella term instead of CDRs encompassing the wealth of medical data availability. We adopt this approach in the present study. As these systems become increasingly ubiquitous, they will decisively contribute as fertile resources for evidence-based clinical practice, patient stratification, and outcome assessment, as well as for data-mining and drug discovery [41]–[45].

Toward this direction, many clinical and research sites have developed such data management and exploration tools to track patient outcomes [46]. Yet, many of them receive limited adoption from the clinical and research communities because they require manual data entry and do not furnish the necessary tools required to enable end-users to perform advanced queries. More recently, there has been a much greater emphasis placed on developing automated extraction, transformation and load (ETL) interfaces. ETLs can accommodate the full spectrum of clinical information, imaging studies and genomic information. Hence, it is possible to interrogate multi-modal data in a systematic manner, guide personalized treatment, refine best practices and provide objective, reproducible insight as to the underlying mechanisms of disease onset and progression [47].

One of the most significant challenges towards establishing enterprise-wide EHRs stems from the fact that a tremendous amount of clinical data are found in unstructured or semi-structured format with a significant number of reports generated at 3^rd^ party laboratories. Many institutions simply scan these documents into images or PDFs so that they can be attached to the patient’s EHR. Other reports arrive in Health Level 7 (HL7) format with the clinical content of the message aggregated into a continuous ASCII (American Standard Code for Information Interchange) string. Unfortunately, such solutions address only the most basic requirements of interoperability by allowing the information to flow into another Healthcare Information Technology (HIT) system; but since the data are not discrete, they cannot be easily migrated into a target relational or document-oriented (non-relational) database.

To effectively incorporate this information into the EHRs and achieve semantic interoperability, it is necessary to develop and optimize software that endorses and relies on interoperability profiles and standards. Such standards are defined by the Integrating the Healthcare Enterprise (IHE), HL7 Fast Healthcare Interoperability Resources (FHIR), and Digital Imaging and Communications in Medicine (DICOM), the latter also extending to medical video communications [48]. Moreover, to adopt clinical terminology coding (e.g., Systemized Nomeclature of Medicine-Clinical Terms (SNOMED CT), International Statistical Classification of Diseases and Related Health Problems (ICD) by the World Health Organization (WHO)). In this fashion, software systems will be in position to reliably extract, process, and share data that would otherwise remain locked in paper-based documents [49]. Importantly, (new) data entry (acquisition) in a standardized fashion underpins extensibility that in turns results in increased statistical power of research studies relying on larger cohorts.

The availability of metadata information is central in unambiguously describing processes throughout the data handling cycle. Metadata underpin medical dataset sharing by providing descriptive information that characterize the underlying data. The latter, can be further capitalized towards joint processing of medical datasets constructed under different context, such as clinical practice, research and clinical trials data [50]. A key medical imaging example concept relevant to metadata usage comes from image retrieval. Traditionally, image retrieval relied on image metadata, such as keywords, tags or descriptions. However, with the advent of machine and deep learning AI solutions (see Section IV), content-based image retrieval (CBIR) systems evolved to exploiting rich contents extracted from images (e.g., imaging, statistical, object features, etc.) stored in a structured manner. Today, querying for other images with similar contents typically relies on a content-metadata similarity metric. Supervised, semi-supervised and unsupervised methods can be applied for CBIR extending across imaging modalities [51].

FAIR guiding principles initiative attempts to overcome (meta) data availability, by establishing a set of recommendations towards constituting (meta) data findable, accessible, interoperable, and reusable (FAIR) [52]. At the same time, privacy-preserving data publishing (PPDP) is an active research area aiming to provide the necessary means for openly sharing data. PPDP objective is to preserve patients’ privacy while achieving the minimum possible loss of information [53]. Sharing such data can increase the likelihood of novel findings and replication of existing research results [54]. To accomplish the anonymization of medical imaging data, approaches such as k-anonymity [55], [56], l-diversity [57] and t-closeness [58] are typically used. Toward this direction, multi-institutional collaboration is quickly becoming the vehicle driving the creation of well-curated and semantically annotated large cohorts that are further enhanced with research methods and results metadata, underpinning reproducible, extensible, and explainable research [59], [60]. From a medical imaging research perspective, the quantitative imaging biomarkers alliance (QIBA) [61] and more recently the image biomarker standardisation initiative (IBSI) [62] set the stage for multi-institution collaboration across imaging modalities. QIBA and IBSI vision is to promote reproducible results emanating from imaging research methods by removing interoperability barriers and adopting software, hardware, and nomeclature standards and guidelines [63]–[66]. Disease specific as well as horizontal examples include the Multi-Ethnic Study of Atherosclerosis (MESA - www.mesa-nhlbi.org), the UK biobank (www.ukbiobank.ac.uk_), the Cancer Imaging Archive (TCIA - www.cancerimagingarchive.net/), the Cancer Genome Atlas (TCGA - https://cancergenome.nih.gov/), and the Alzheimer’s Disease Neuroimaging Initiative (ADNI - http://adni.loni.usc.edu/). In a similar context, the CANDLE project (CANcer Distributed Learning Environment) focuses on the development of open-source AI-driven predictive models under a single scalable deep neural network umbrella code. Exploiting the ever-growing volumes and diversity of cancer data and leveraging exascale computing capabilities, it aspires to advance and accelerate cancer research.

The co-localization of such a broad number of correlated data elements representing a wide spectrum of clinical information, imaging studies, and genomic information, coupled with appropriate tools for data mining, are instrumental for integrative analytics approaches and will lead to unique opportunities for improving precision medicine [67], [68].

## IV. Processing, Analysis, and Understanding in Radiology

This section reviews the general field of image analysis and understanding in radiology whereas a similar approach is portrayed in the next section for digital pathology.

Medical image analysis typically involves the delineation of the objects of interest (segmentation) or description of labels (classification) [69]–[72]. Examples include segmentation of the heart for cardiology and identification of cancer for pathology. To date, medical image analysis has been hampered by a lack of theoretical understanding on how to optimally choose and process visual features. A number of *ad hoc* (or *hand-crafted*) feature analysis approaches have achieved some success in different applications, by explicitly defining a prior set of features and processing steps. However, no single method has provided robust, cross-domain application solutions. The recent advent of machine learning approaches has provided good results in a wide range of applications. These approaches, attempt to learn the features of interest and optimize parameters based on training examples. However, these methods are often difficult to engineer since they can fail in unpredictable ways and are subject to bias or spurious feature identification due to limitations in the training dataset. An important mechanism for advancing the field is by open access challenges in which participants can benchmark methods on standardized datasets. Notable examples of challenges include dermoscopic skin lesions [73], brain MRI [74], [75], heart MRI [76], quantitative perfusion [77], classification of heart disease from statistical shape models [78], retinal blood vessels segmentation [79], [80], general anatomy (i.e., the VISCERAL project evaluated the subjectivity of 20 segmentation algorithms [81]), segmentation of several organs together (the decathlon challenge) [82], and many others. An up-to-date list of open and ongoing biomedical challenges appears in [83]. These challenges have provided a footing for advances in medical image analysis and helped push the field forward; however, a recent analysis of challenge design has showed that biases exist that questions how easy would be to translate methods to clinical practice [84].

### A. Feature Analysis

There has been a wealth of literature on medical image analysis using signal analysis, statistical modelling, etc. [71]. Some of the most successful include multi-atlas segmentation [85], graph cuts [86], and active shape models [87], [88]. Multi-atlas segmentation utilizes a set of labelled cases (atlases) which are selected to represent the variation in the population. The image to be segmented is registered to each atlas (i.e., using voxel-based morphometry [89]) and the propagated labels from each atlas are fused into a consensus label for that image. This procedure adds robustness since errors associated with a particular atlas are averaged to form a maximum likelihood consensus. A similarity metric can then be used to weight the candidate segmentations. A powerful alternative method attempts to model the object as a deformable structure, and optimize the position of the boundaries according to a similarity metric [87]–[90]. Active shape models contain information on the statistical variation of the object in the population and the characteristic of their images [91]. These methods are typically iterative and may thus get stuck in a local minimum. On the other hand, graph cut algorithms facilitate a global optimal solution [86]. Despite the initial graph construction being computationally expensive, updates to the weights (interaction) can be computed in real time.

### B. Machine Learning

Machine learning (prior to deep learning which we analyse below) involves the definition of a learning problem to solve a task based on inputs [92]. To reduce data dimensionality and induce necessary invariances and covariances (e.g. robustness to intensity changes or scale) early machine learning approaches relied on hand-crafted features to represent data. In imaging data several transforms have been used to capture local correlation and disentangle frequency components spanning from Fourier, Cosine or Wavelet transform to the more recent Gabor filters that offer also directionality of the extracted features and superior texture information (when this is deemed useful for the decision). In an attempt to reduce data dimensionality or to learn in a data-driven fashion features, Principal and Independent Component Analyses have been used and [93] also the somewhat related (with some assumptions) K-means algorithm [94]. These approaches formulate feature extraction within a reconstruction objective imposing different criteria on the reconstruction and the projection space (e.g. PCA assumes the projection space is orthogonal). Each application then required a significant effort in identifying the proper features (known as feature engineering), which would then be fed into a learnable decision algorithm (for classification or regression). A plethora of algorithms have been proposed for this purpose, a common choice being support vector machines [95], due to the ease of implementation and the well understood nonlinear kernels. Alternatively, random forest methods [96] employ an ensemble of decision trees, where each tree is trained on a different subset of the training cases, improving the robustness of the overall classifier. An alternative classification method is provided by probabilistic boosting trees [97], which forms a binary tree of strong classifiers using a boosting approach to train each node by combining a set of weak classifiers. However, recent advances in GPU processing and availability of data for training have led to a rapid expansion in neural nets and deep learning for regression and classification [98]. Deep learning methods instead optimize simultaneously for the decision (classification or regression) whilst identifying and learning suitable input features. Thus, in lieu of feature engineering, learning how to represent data and how to solve for the decision are now done in a completely data-driven fashion, notwithstanding the existence of approaches combining feature-engineering and deep learning [99]. Exemplar deep learning approaches for medical imaging purpose are discussed in the next subsections.

### C. Deep Learning for Segmentation

One of the earliest applications of convolutional neural networks (CNN, the currently most common form of deep learning) has appeared as early as 1995, where a CNN was used for lung nodule detection in chest x-rays [100]. Since then, fueled by the revolutionary results of AlexNet [101] and incarnations of patch-based adaptations of Deep Boltzmann Machines and stacked autoencoders, deep learning based segmentation of anatomy and pathology has witnessed a revolution (see also Table II), where for some tasks now we observe human level performance [102]. In this section, we aim to analyse key works and trends in the area, while we point readers to relevant, thorough reviews in [69], [70].

The major draw of deep learning and convolutional architectures is the ability to learn suitable features and decision functions in tandem. While AlexNet quickly set the standard for classification (that was profusely adapted also for classification of medical tasks, see next subsection) it was the realisation that dense predictions can be obtained from classification networks by convolutionalization that enabled powerful segmentation algorithms [103]. The limitations of such approaches for medical image segmentation were quickly realised and led to the discovery of U-Net [104], which is even today one of the most successful architectures for medical image segmentation.

The U-Net is simple in its conception: an encoder-decoder network that goes through a bottleneck but contains skip connections from encoding to decoding layers. The skip connections allow the model to be trained even with few input data and offer highly accurate segmentation boundaries, albeit perhaps at the “loss” of a clearly determined latent space. While the original U-Net was 2D, in 2016, the 3D U-net was proposed that allowed full volumetric processing of imaging data [105], maintaining the same principles of the original U-net.

Several works were inspired by treating image segmentation as an image-to-image translation (and synthesis) problem. This introduced a whole cadre of approaches that permit for unsupervised and semi-supervised learning working in tandem with adversarial training [106] to augment training data leveraging label maps or input images from other domains. The most characteristic examples are works inspired by CycleGAN [107]. CycleGAN allows mapping of one image domain to another image domain even without having pairs of images. Early on Chartsias *et al.*, used this idea to generate new images and corresponding myocardial segmentations mapping CT to MRI images [108]. Similarly, Wolterink *et al.* used it in the context of brain imaging [109]. Both these approaches paired and unpaired information (defining a pair as an input image and its segmentation) differently to map between different modalities (MR to CT) or different MR sequences.

Concretely rooted in the area of semi-supervised learning [110] are approaches that use discriminators to approximate distributions of shapes (and thus act as shape priors), to solve the segmentation task in an unsupervised manner in the heart or the brain [111]. However, in the context of cardiac segmentation, the work of Chartsias *et al.*, showed that when combined with auto-encoding principles and factorised learning, a shape-prior aided with reconstruction objectives offer a compelling solution to semi-supervised learning for myocardial segmentation [112].

We highlight that all the above works treat expert delineations as ground truth, whereas our community is well aware of the variability in the agreement between experts in delineation tasks. Inspired by aforementioned, Kohl *et al.* devised a probabilistic U-Net, where the network learns from a variety of annotations without need to provide (externally) a consensus [113]. However, we note that use of supervision via training exemplars as a signal could be limited and may not fully realize the potential of deep learning.

### D. Deep Learning for Classification

Deep learning algorithms have been extensively used for disease classification, or screening, and have resulted in excellent performance in many tasks (see Table II). Applications include screening for acute neurologic events [114], diabetic retinopathy [115], and melanoma [116].

Like segmentation, these classification tasks have also benefited from CNNs. Many of the network architectures that have been proven on the ImageNet image classification challenge [117] have seen reuse for medical imaging tasks by fine-tuning previously trained layers. References [118] and [119] were among the first that assessed the feasibility of using CNN-based models trained on large natural image datasets, for medical tasks. In [118], the authors showed that pre-training a model on natural images and fine-tuning its parameters for a new medical imaging task gave excellent results. These findings were reinforced in [120] to demonstrate that fine-tuning a pre-trained model generally performs better than a model trained from scratch. Ensembles of pre-trained models can also be fine-tuned to achieve strong performance as demonstrated in [121].

This transfer learning approach is not straightforward, however, when the objective is tissue classification of 3D image data. Here, transfer learning from natural images is not possible without first condensing the 3D data into two dimensions. Practitioners have proposed a myriad of choices on how to handle this issue, many of which have been quite successful. Alternative approaches directly exploit the 3D data by using architectures that perform 3D convolutions and then train the network from scratch on 3D medical images [122]–[126]. Other notable techniques include slicing 3D data into different 2D views before fusing to obtain a final classification score [127]. Learning lung nodule features using a 2D autoencoder [128] and then employing a decision tree for distinguishing between benign nodules and malignant ones was proposed in [129].

Development of an initial network–in which transfer learning is dependent – is often difficult and time-consuming. Automated Machine Learning (AutoML) has eased this burden by finding optimal networks hyperparameters [130] and, more recently, optimal network architectures [131]. We suspect these high-level training paradigms will soon impact medical image analysis.

Overall, irrespective of the training strategy used, classification tasks in medical imaging are dominated by some formulation of a CNN – often with fully-connected layers at the end to perform the final classification. With bountiful training data, CNNs can often achieve state-of-the-art performance; however, deep learning methods generally suffer with limited training data. As discussed, transfer learning has been beneficial in coping with scant data, but the continued availability of large, open datasets of medical images will play a big part in strengthening classification tasks in the medical domain.

### E. CNN Interpretability

Although Deep CNNs have achieved extremely high accuracy, they are still black-box functions with multiple layers of nonlinearities. It is therefore essential to trust the output of these networks and to be able to verify that the predictions are from learning appropriate representations, and not from overfitting the training data. Deep CNN interpretability is an emerging area of machine learning research targeting a better understanding of what the network has learned and how it derives its classification decisions. One simple approach consists of visualizing the nearest neighbors of image patches in the fully connected feature space [101]. Another common approach that is used to shed light on the predictions of Deep CNN is based on creating saliency maps [132] and guided backpropagation [133], [134]. These approaches aim to identify voxels in an input image that are important for classification based on computing the gradient of a given neuron at a fixed layer with respect to voxels in the input image. Another similar approach, that is not specific to an input image, uses gradient ascent optimization to generate a synthetic image that maximally activates a given neuron [135]. Feature inversion, where the difference between an input image and its reconstruction from a representation at a given layer, is another approach that can capture the relevant patches of the image at the considered layer [136]. Other methods for interpreting and understanding deep networks can be found in [137]–[139]. Specifically, for medical imaging, techniques described in [140] interpret predictions in a visually and semantically meaningful way while task-specific features in [141] are developed such that their deep learning system can make transparent classification predictions. Another example uses multitask learning to model the relationship between benign-malignant and eight other morphological attributes in lung nodules with the goal of an interpretable classification [142]. Importantly, due diligence must be done during the design of CNN systems in the medical domain to ensure spurious correlations in the training data are not incorrectly learned.

### F. Interpretation and Understanding

Once object geometry and function has been quantified, patient cohorts can be studied in terms of the statistical variation of shape and motion across large numbers of cases. In the Multi-Ethnic Study of Atherosclerosis, heart shape variations derived from MRI examinations were associated with known cardiovascular risk factors [143]. Moreover, application of imaging informatics methodologies in the cardiovascular system has produced important new knowledge and has improved our understanding of normal function as well as of pathophysiology, diagnosis and treatment of cardiovascular disorders [144]. In the brain, atlas-based neuroinfoimatics enables new information on structure to predict neurodegenerative diseases [145].

At the same time, it is also possible to extract information on biophysical parameters of tissues and organs from medical imaging data. For example, in elastography, it is possible to estimate tissue compliance from the motion of wave imaged using ultrasound or MRI [146], whereas in the heart, myocardial stiffness is associated with disease processes. Given knowledge of the boundary loading, and imaged geometry and displacements, finite element analysis can estimate material properties compatible with the imaged deformation [147].

## V. Processing, Analysis, and Understanding in Digital Pathology

Pathology classifications and interpretations have traditionally been developed through pathologist examination of tissue prepared on glass slides using microscopes. Analyses of single tissue and TMA images have the potential to extract highly detailed and novel information about the morphology of normal and diseased tissue and characterization of disease mechanics at the sub-cellular scale. Studies have validated and shown the value of digitized tissue slides in biomedical research [148]–[152]. Whole slide images can contain hundreds of thousands or more cells and nuclei. Detection, segmentation and labeling of slide tissue image data can thus lead to massive, information rich datasets. These datasets can be correlated to molecular tumor characteristics and can be used to quantitatively characterize tissue at multiple spatial scales to create biomarkers that predict outcome and treatment response [150], [152]–[154]. In addition, multiscale tissue characterizations can be employed in epidemiological and surveillance studies. The National Cancer Institute SEER program is exploring the use of whole slide imaging extracted features to add cancer biology phenotype data to its surveillance efforts. Digital pathology has made great strides in the past 20 years. A good review of challenges and advancements in digital pathology is provided in several publications [155]–[157]. Whole slide imaging is also now employed at some sites for primary anatomic pathology diagnostics. In light of advances in imaging instruments and software, the FDA approved in 2017 the use of a commercial digital pathology system in clinical settings [158]. A summary of AI-based medical imaging systems that have obtained FDA approval appear in Table III.

### A. Segmentation and Classification

Routine availability of digitized pathology images, coupled with well-known issues associated with inter-observer variability in how pathologists interpret studies [159], has led to increased interest in computer-assisted decision support systems. Image analysis algorithms, however, have to tackle several challenges in order to efficiently, accurately and reliably extract information from tissue images. Tissue images contain a much denser amount of information than many other imaging modalities, encoded at multiple scales (pixels, objects such as nuclei and cells, and regions such as tumor and stromal tissue areas). This is further compounded by heterogeneity in structure and texture characteristics across tissue specimens from different disease regions and subtypes. A major challenge in pathology decision support also arises from the complex and nuanced nature of many pathology classification systems. Classifications can hinge of the fraction of the specimen found to have one or another pattern of tissue abnormality. In such cases, the assessment of abnormality and the estimate of tissue area are both subjective. When interpretation could only be carried out using glass slides, the profound way of reducing inter-observer variability was for multiple pathologists to view the same glass slides and to confer on interpretation. These challenges have motivated many efforts for the development of image analysis methods to automate whole slide image pathology interpretation. While few of these methods have found their way into clinical practice, results are promising and seem almost certain to ultimately lead to the development of effective methods to routinely provide algorithmic anatomic pathology second opinions. A comprehensive review of these initiatives appears in [160]–[162].

Some of the earlier works employed statistical techniques and machine learning algorithms to segment and classify tissue images. Bamford and Lovell, for example, used active contours to segment nuclei in Pap stained cell images [163]. Malpica *et al.* applied watershed-based algorithms for separation of nuclei in cell clusters [164]. Kong *et al.* utilized a combination of grayscale reconstruction, thresholding, and watershed-based methods [165]. Gao *et al.* adapted a hierarchical approach based on mean-shift and clustering analysis [166]. Work by Al-Kofahi *et al.* implemented graph-cuts and multiscale filtering methods to detect nuclei and delineate their boundaries [167]. In recent years, deep learning methods have rapidly grown in importance in pathology image analysis [160]. Deep learning approaches make it possible to automate many aspects of the information extraction and classification process. A variety of methods have been developed to classify tissue regions or whole slide images, depending on the context and the disease site. Classifications can hinge on whether regions of tissue contain tumor, necrosis or immune cells. Classification can also target algorithmic assessment of whether tissue regions are consistent with pathologist descriptions of tissue patterns. An automated system for the analysis of lung adenocarcinoma based on nuclear features and WHO subtype classification using deep convolutional neural networks and computational imaging signatures was developed, for example, in [168]. There has been a wealth of work over the past twenty years to classify histological patterns in different disease sites and cancer types (e.g. Gleason Grade in prostate cancer, lung cancer, breast cancer, melanoma, lymphoma and neuroblastoma) using statistical methods and machine and deep learning techniques [154], [169], [170].

Detection of cancer metastases is an important diagnostic problem to which machine-learning methods have been applied. The CAMELYON challenges target methods for algorithmic detection and classification of breast cancer metastases in H&E whole slide lymph node sections [171]. The best performing methods employed convolutional neural networks differing in network architecture, training methods, and methods for pre- and post- processing. Overall, there has been ongoing improvement in performance of algorithms that detect, segment and classify cells and nuclei. These algorithms often form crucial components of cancer biomarker algorithms. Their results are used to generate quantitative summaries and maps of the size, shape, and texture of nuclei as well as statistical characterizations of spatial relationships between different types of nuclei [172]–[176]. One of the challenges in nuclear characterization is to generalize the task across different tissue types. This is especially problematic because generating ground truth datasets for training is a labor intensive and time-consuming process and requires the involvement of expert pathologists. Deep learning generative adversarial networks (GANs) have proved to be useful in generalizing training datasets in that respect [177].

### B. Interpretation and Understanding

There is increasing attention paid to the role of tumor immune interaction in determining outcome and response to treatment. In addition, immune therapy is increasingly employed in cancer treatment. High levels of lymphocyte infiltration have been related to longer disease-free survival or improved overall survival (OS) in multiple cancer types [178] including early stage triple-negative and HER2-positive breast cancer [179]. The spatial distribution of lymphocytes with respect to tumor, tumor boundary and tumor associated stroma are also important factors in cancer prognosis [180]. A variety of recent efforts relies on deep learning algorithms to classify TIL regions in H&E images. One recent effort targeted characterization of TIL regions in lung cancer, while another, carried out in the context of TCGA Pan Cancer Immune group, looked across tumor types to correlate deep learning derived spatial TIL patterns with molecular data and outcome. A 3^rd^ study employed a structured crowd sourcing method to generate tumor infiltrating lymphocyte maps [152], [181]. These studies showed there are correlations between characterizations of TIL patterns, as analyzed by computerized algorithms, and patient survival rates and groupings of patients based on subclasses of immunotypes. These studies demonstrate the value of whole slide tissue imaging in producing quantitative evaluations of sub-cellular data and opportunities for richer correlative studies.

Although there has been some progress made in the development of automated methods for assessing TMA images, most of systems are limited by the fact that they are closed and proprietary; do not exploit the potential of advanced computer vision techniques; and/or do not conform with emerging data standards. In addition to the significant analytical issues, the sheer volume of data, text, and images arising from even limited studies involving tissue microarrays pose significant computational and data management challenges (see also Section VI.B). Tumor expression of immune system-related proteins may reveal the tumor immune status which in turn can be used to determine the most appropriate choices for immunotherapy. Objective evaluation of tumor biomarker expression is needed but often challenging. For instance, human leukocyte antigen (HLA) class I tumor epithelium expression is difficult to quantify by eye due to its presence on both tumor epithelial cells and tumor stromal cells, as well as tumor-infiltrating immune cells [182].

To maximize the flexibility and utility of the computational imaging tools that are being developed, it will be necessary to address the challenge of batch affect, which arises due to the fact that histopathology tissue slides from different institutions show heterogeneous appearances as a result of differences in tissue preparation and staining procedures. Prediction models had been investigated as a means for reliably learning from one domain to map into a new domain directly. This was accomplished by introducing unsupervised domain adaptation to transfer the discriminative knowledge obtained from the source domain to the target domain without requiring re-labeling images at the target domain [183]. This paper has focused on analysis of Hematoxylin and Eosin (H&E) stained tissue images. H&E is one of the main tissue stains and is most commonly used stain in histopathology. Tissue specimens taken from patients are routinely stained with H&E for evaluation by pathologists for cancer diagnosis. There is a large body of image analysis research that targets H&E stained tissue as covered in this paper. In research and clinical settings other types of staining and imaging techniques, such as fluorescence microscopy and immunohistochemical techniques, are also employed [184]–[185]. These staining techniques can be used to boosting signal specific morphological features of tissue –e.g., emphasizing proteins and macromolecules in cells and tissue samples. An increasing number of histopathology imaging projects are targeting methods for analysis of images obtained from fluorescence microscopy and immunostaining techniques (e.g., [186]–[192]).

## VI. Visualization and Navigation

### A. Biomedical 3D Reconstruction and Visualization

Three-dimensional (3D) reconstruction concerns the detailed 3D surface generation and visualization of specific anatomical structures, such as arteries, vessels, organs, body parts and abnormal morphologies e.g. tumors, lesions, injuries, scars and cysts. It entails meshing and rendering techniques are used for completing the seamless boundary surface, generating the volumetric mesh, followed by smoothing and refinement. By enabling precise position and orientation of the patient’s anatomy, 3D visualization can contribute to the design of aggressive surgery and radiotherapy strategies, with realistic testing and verification, with extensive applications in spinal surgery, joint replacement, neuro-interventions, as well as coronary and aortic stenting [193]. Furthermore, 3D reconstruction constitutes the necessary step towards biomedical modeling of organs, dynamic functionality, diffusion processes, hemodynamic flow and fluid dynamics in arteries, as well as mechanical loads and properties of body parts, tumors, lesions and vessels, such as wall / shear stress and strain and tissue displacement [194].

In medical imaging applications with human tissues, registration of slices must be performed in an elastic form [195]. To that respect, feature-based registration appears more suitable in the case of vessels’ contours and centerline [196], while the intensity-based registration can be effectively used for image slices depicting abnormal morphologies such as tumors [197]. The selection of appropriate meshing and rendering techniques highly depends on the imaging modality and the corresponding tissue type. To this respect, Surface Rendering techniques are exploited for the reconstruction of 3D boundaries and geometry of arteries and vessels through the iso-contours extracted from each slice of intravascular ultrasound or CT angiography. Furthermore, NURBS are effectively used as a meshing technique for generating and characterizing lumen and media-adventitia surfaces of vascular geometric models, such as aortic, carotid, cerebral and coronary arteries, deployed for the reconstruction of aneurysms and atherosclerotic lesions [196], [198]. The representation of solid tissues and masses, i.e. tumors, organs and body parts, is widely performed by means of Volume Rendering techniques, such as ray-casting, since they are capable of visualizing the entire medical volume as a compact structure but also with great transparency, even though they might be derived from relatively low contrast image data.

The reconstruction process necessitates expert knowledge and guidance. However, this is particularly time consuming and hence not applicable in the analysis of larger numbers of patient-specific cases. For those situations, automatic segmentation and reconstruction systems are needed. The biggest problem with automatic segmentation and 3D reconstruction is the inability to fully automate the segmentation process, because of different imaging modalities, varying vessel geometries, and the quality of source images [199]. Processing of large numbers of images require fast algorithms for segmentation and reconstruction. There are several ways to overcome this challenge such as parallel algorithms for segmentation and application of neural networks as discussed in Sections IV–V, the use of multiscale processing techniques, as well as the use of multiple computer systems where each system works on an image in real time.

### B. Data Management, Visualization and Processing in Digital Pathology

Digital pathology is an inherently interactive human-guided activity. This includes labeling data for algorithm development, visualization of images and features for tuning algorithms, as well as explaining findings, and finally gearing systems towards clinical applications. It requires interactive systems that can query the underlying data and feature management systems, as well as support interactive visualizations. Such interactivity is a prerequisite to wide-scale adoption of digital pathology in imaging informatics applications. There are a variety of open source systems that support visualization, management, and query of features, extracted from whole slide images along with the generation of whole slide image annotations and markups. One such system is the QuIP software system [201]. QuIP is an open-source system that uses the caMicroscope viewer [202] to support the interactive visualization of images, image annotations, and segmentation results as overlays of heatmaps or polygons. QuIP includes FeatureScape - a visual analytic tool that supports interactive exploration of feature and segmentation maps. Other open-source systems that carry out these or related tasks are QuPath [203], the Pathology Image Informatics Platform (PIIP) for visualization, analysis, and management [204], the Digital Slide Archive (DSA) [205] and Cytomine [206]. These platforms are designed for local (QuPath, PIIP) or web-based (QuIP, caMicroscope, DSA) visualization, management and analysis of whole slide images. New tools and methods are also being developed to support knowledge representation and indexing of imaged specimens based on advanced feature metrics. These metrics include computational biomarkers with similarity indices that enable rapid search and retrieval of similar regions of interest from large datasets of images. Together, these technologies will enable investigators to conduct high-throughput analysis of tissue microarrays composed of large patient cohorts, store and mine large data sets and generate and test hypotheses [200].

The processing of digital pathology images is a challenging activity, in part due to the size of whole-slide images, but also because of an abundance of image formats and the frequent need for human guidance and intervention during processing. There are some efforts towards the adoption of DICOM in digital pathology, including the availability of tools such as the Orthanc DICOMizer [207] that can convert a pyramidal tiled tiff file into a DICOM pathology file. caMicroscope [202] supports the visualization of DICOM pathology files over the DICOMWeb API [208]. These efforts are few and far between, and most solutions adopt libraries such as OpenSlide [209] or Bio-Formats [210] to navigate the plethora of open and proprietary scanner formats. Digital pathology algorithms work well with high resolution images to extract detailed imaging features from tissue data. Since digital pathology images can grow to a few GBs, compressed, per-image, the local processing of digital pathology images can be severely affected by the computational capacity of an interactive workstation. In such cases, some algorithms can work on regions of interest (ROI) identified by a user or on lower-resolution, down-sampled images. The growing popularity of containerization technologies such as Docker [211] has opened a new mechanism to distribute algorithms and pathology pipelines. There is also growing interest in the use of cloud computing for digital pathology, driven by the rapid decline in costs, making them increasingly cost-effective solutions for large-scale computing. A number of groups, predominantly in the genomics community, have developed solutions for deploying genomic pipelines on the cloud [212]–[214]. QuIP includes cloud-based pipelines for tumor infiltrating lymphocyte analysis and nuclear segmentation. These are available as APIs and deployed as containers as well as pipelines in workflow definition language (WDL) using a cross-platform workflow orchestrator, which supports multiple cloud and high performance computing (HPC) platforms. The work in this area is highly preliminary, but one that is likely to see widespread adoption in the forthcoming years. Applications include algorithm validation, deployment of algorithms in clinical studies and clinical trials, and algorithm development particularly in systems that employ transfer learning.

### C. In Silico Modeling of Malignant Tumors

**Fig. 2.**
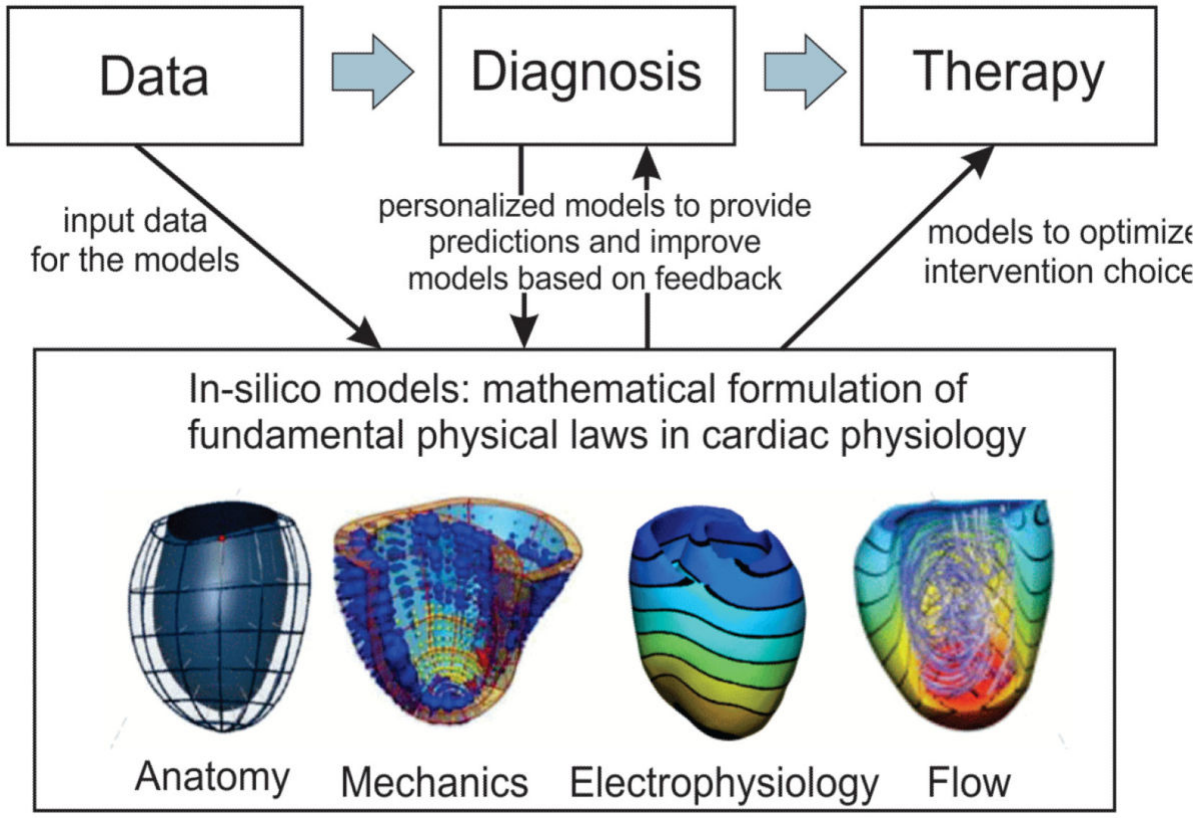
In silico modelling paradigm of cardiovascular disease with application to heart.

Applications of in-silico models evolve drastically in early diagnosis and prognosis, with personalized therapy planning, noninvasive and invasive interactive treatment, as well as planning of pre-operative stages, chemotherapy and radiotherapy (see Fig. 2). The potential of inferring reliable predictions on the macroscopic tumor growth is of paramount importance to the clinical practice, since the tumor progression dynamics can be estimated under the effect of several factors and the application of alternative therapeutic schemes. Several mathematical and computational models have been developed to investigate the mechanisms that govern cancer progression and invasion, aiming to predict its future spatial and temporal status with or without the effects of therapeutic strategies.

Recent efforts towards in silico modeling focus on multi-compartment models for describing how subpopulations of various cell types proliferate and diffuse, while they are computationally efficient. Furthermore, multiscale approaches link in space and time the interactions at different biological levels, such as molecular, microscopic cellular and macroscopic tumor scale [215]. Multi-compartment approaches can reflect the macroscopic volume expansion while they reveal particular tumor aspects, such as the spatial distributions of cellular densities of different phenotypes taking into account tissue heterogeneity and anisotropy issues, as well as the chemical microenvironment with the available nutrients [216]. The metabolic influence of oxygen, glucose and lactate is incorporated in multi-compartment models of tumor spatio-temporal evolution, enabling the formation of cell populations with different metabolic profile, proliferation and diffusion rates. Methodological limitations of such approaches relate mainly to reduced ability of simulating specific cellular factors (e.g. cell to cell adhesion) and subcellular-scale processes [217], which play an important role in regulating cellular behavior and determine tumor expansion/metastasis.

Recent trends in modeling seek to incorporate the macroscopic tumor progress along with dynamic changes of chemical ingredients (such as glucose, oxygen, chemotherapeutic drugs, etc), but also the influence of individual cell expressions resulting from the intracellular signaling cascades and gene characteristics. Along this direction, multiscale cancer models allow to link in space and time the different biological scales affecting the macroscopic tumor development. They facilitate model development in precision medicine under the 3R principles of in vivo experimentation related to replacement, reduction and refinement [218] of experimentation on life samples. Distinct spatial and temporal scales have been considered, such as the subcellular scale of molecular pathways and gene expressions, the microscopic-cellular level of individual cell’s behavior and phenotypic properties, the microenvironmental scale of the diffusing chemical ingredients, the tissue-multicellular extent of different cell-regions and the macroscopic scale of the tumor volume. The interconnection of the different levels is considered great challenge of in-silico models, through coupling of blood flow, angiogenesis, vascular remodeling, nutrient transport and consumption, as well as movement interactions between normal and cancer cells [219].

Despite the progress, challenging issues still remain in cancer growth models. Important factors include the ability to simulate tumor microenvironment, as well as cell-to-cell interactions, the effectiveness of addressing body heterogeneity and anisotropy issues with diffusion tensors, the potential of engaging the dynamically changing metabolic profile of tumor, and the ability of including interactions on cancer growth at biomolecular level, considering gene mutations and malignancy of endogenous receptors.

### D. Digital Twins

In general, digital twin uses and applications benefit not only from CAD reconstruction tools but also engage dynamic modelling stemming from either theoretical developments or real-life measurements merging the Internet of Things with artificial intelligence and data analytics [220]–[221]. In this form, the digital equivalent of a complex human functional system enables the consideration of event dynamics, such as tumour growth or information transfer in epilepsy network, as well as a systemic response to therapy, such as response to pharmacogenomics or targeted radiotherapy [222].

Since the digital twin can incorporate modelling at different resolutions, from organ structure to cellular and genomic level, it may enable complex simulations [223] with the use of AI tools to integrate huge amounts of data and knowledge aiming at improved diagnostics and therapeutic treatments, without harming the patient. Furthermore, such a twin can also act as a framework to support human-machine collaboration in testing and simulating complex invasive operations without even engaging the patient.

## VII. Integrative Analytics

### A. Medical Imaging in the Era of Precision Medicine

**Fig. 3.**
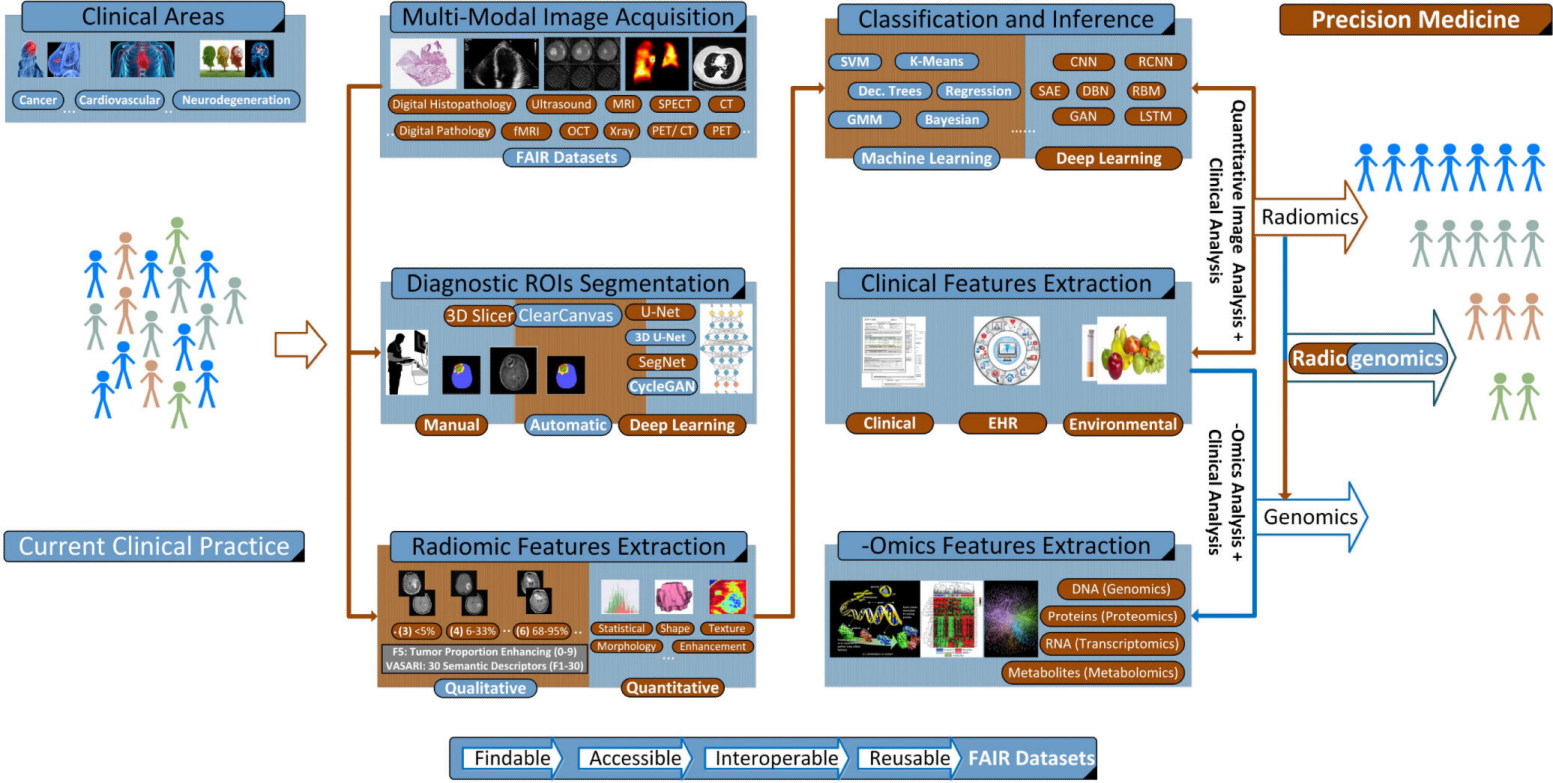
Radiogenomics System Diagram: An abstract system diagram demonstrating the use of radiogenomics approaches in the context of precision medicine [68]. Based on the clinical case, (multi-modal) image acquisition is performed. Then, manual and/or automatic segmentation of the diagnostic regions of interest follows, driving quantitative and/or qualitative radiomic features extraction and machine learning approaches for segmentation, classification and inference. Alternatively, emerging deep learning methods using raw pixel intensities can be used for the same purpose. Radiogenomics approaches investigate the relationships between imaging and genomic features and how radiomics and genomics signatures, when processed jointly, can better describe clinical outcomes. On the other hand, radiomics research is focused on characterizing the relationship between quantitative imaging and clinical features.

Radiologists and pathologists are routinely called upon to evaluate and interpret a range of macroscopic and microscopic images to render diagnoses and to engage in a wide range of research activities. The assessments that are made ultimately lead to clinical decisions that determine how patients are treated and predict outcomes. Precision medicine is an emerging approach for administering healthcare that aims to improve the accuracy with which clinical decisions are rendered towards improving the delivery of personalized treatment and therapy planning for patients as depicted in Fig. 3 [67]. In that context, physicians have become increasingly reliant upon sophisticated molecular and genomic tests, which can augment standard pathology and radiology practices in order to refine stratification of patient populations and manage individual care. Recent advances in computational imaging, clinical genomics and high-performance computing now make it possible to consider multiple combinations of clinico-pathologic data points, simultaneously. Such advances provide unparalleled insight regarding the underlying mechanisms of disease progression and could be used to develop a new generation of diagnostic and prognostic metrics and tools. From a medical imaging perspective, radiogenomics paradigm integrates afore-described objectives towards advancing precision medicine.

### B. Radiogenomics for Integrative Analytics

Radiomics research has emerged as a non-invasive approach of significant prognostic value [224]. Through the construction of imaging signatures (i.e., fusing shape, texture, morphology, intensity, etc., features) and their subsequent association to clinical outcomes, devising robust predictive models (or quantitative imaging biomarkers) is achieved [225]. Incorporating longitudinal and multi-modality radiology and pathology (see also Section VII.C) image features further enhances the discriminatory power of these models. A dense literature demonstrates the potentially transforming impact of radiomics for different disease staging such as cancer, neurodegenerative, and cardiovascular diseases [224]–[228]. Going one-step further, radiogenomics methods extend radiomics approaches by investigating the correlation between, for example, a tumor’s characteristics in terms of quantitative imaging features and its molecular and genetic profiling [68]. A schematic representation of radiomic and radiogenomics approaches appears in Fig. 3.

During the transformation from a benign to malignant state and throughout the course of disease progression, changes occur in the underlying molecular, histologic and protein expression patterns, with each contributing a different perspective and complementary strength. Clearly then, the objective is to generate surrogate imaging biomarkers connecting cancer phenotypes to genotypes, providing a powerful and yet non-invasive prognostic and diagnostic tool in the hands of physicians. At the same time, the joint development of radiogenomic signatures, involves the integrated mining of both imaging and -omics features, towards constructing robust predictive models that better correlate and describe clinical outcomes, as compared with imaging, genomics or histopathology alone [68].

The advent of radiogenomics research is closely aligned with associated advances in inter- and multi- institutional collaboration and the establishment of well curated, FAIR-driven repositories that encompass the substantial amount of semantically annotated (big) data, underpinning precision medicine (see Section III). Such example is the TCIA and the TCGA repositories, which provide matched imaging, genetic and clinical data for over 20 different cancer types. Importantly, these data further facilitate consensus ratings on radiology images (e.g., MRI) of expert radiologists to alleviate inconsistencies that often arise due to subjective impressions and inter- and intra-observer variability [229]. Moreover, driven by the observation that objectivity and reproducibility improve when conclusions are based upon computer-assisted decision support [230]–[233], research initiatives from TCIA groups attempt to formalize methodological processes thus accommodating extensibility and explainability.

#### 1) The TCIA/ TCGA Initiatives Paradigm:

The breast and glioma phenotype groups in TCIA, investigating *breast invasive carcinoma* (BRCA) and *glioblastoma* (GBM) and *lower grade glioma* (LGG), respectively, are examples of such initiatives. In this sequence, the breast phenotype group defined a total of 38 radiomics features driving reproducible radiogenomics research hypothesis testing [234]. Stemming from T1-weighted Dynamic Contrast Enhancement (DCE) MRI, radiomics features are classified into six phenotype categories, namely: (i) *size (4)*, (ii) *shape (3)*, (iii) *morphology (3)*, (iv) *enhancement texture (14)*, (v) *kinetic curve (10)*, and (vi) *enhancement-variance kinetics (4)*. Likewise, the glioma phenotype group relies on the VASARI feature set to subjectively interpret MRI visual cues. VASARI is a reference consensus schema composed of 30 descriptive features classified with respect to (i) *non-enhanced tumor*, (ii) *contrast-enhanced tumor*, (iii) *necrosis*, and (iv) *edema*. VASARI is widely used in corresponding radiogenomics studies driving the quantitative imaging analysis from a clinical perspective [235]. In terms of genetic analysis, features are extracted from the TCGA website, using enabling software such as the TCGA-Assembler.

Breast phenotype group studies documented significant associations between specific radiomics features (e.g., size and enhancement texture) and breast tumor staging. Moreover, they performed relatively well in predicting clinical receptor status, multigene assay recurrence scores (poor vs good prognosis), and molecular subtyping. Imaging phenotypes where further associated with miRNA and protein expressions [236]–[239].

At the same time, hypothesis testing in glioma phenotype group verified the significant association between certain radiomic and genomic features with respect to overall and progression free survival, while joint radiogenomic signatures were found to increase the predictive ability of generated models. Importantly, imaging features were linked to molecular GBM subtype classification (based on Verhaak and/ or Philips classification) providing for non-invasive prognosis [68], [240].

#### 2) Deep Learning Based Radiogenomics:

While still at its infancy, relying mostly on transfer learning approaches, deep learning methods are projected to expand and transform radiomics and radiogenomics research. Indicative studies focusing on cancer research involve discriminating between Luminal A and other molecular subtypes for breast cancer [241], predicting bladder cancer treatment response [242], IDH1 mutation status for LGG [243], [244], and MGMT methylation status for GBM [245], as well as predicting overall survival for GBM patients [246] and non-disease specific subjects [247].

### C. Integrative Analytics in Digital Pathology

Recently, the scope of image-based investigations has expanded to include synthesis of results from pathology images, genome information and correlated clinical information. For example a recent set of experiments utilized 86 breast cancer cases from the Genomics Data Commons (GDC) repository to demonstrate that using a combination of image- based and genomic features served to improve classification accuracy significantly [248]. Other work demonstrated the potential of utilizing a combination of genomic and computational imaging signatures to characterize prostate cancer. The results of the study show that integrating image biomarkers from CNN with a recurrence network model, called long short-term memory LSTM and genomic pathway scores, is more strongly correlated with a patient’s recurrence of disease as compared to using standard clinical markers and image-based texture features [249]. An important computational issue is how to effectively integrate the omics data with digitized pathology images for biomedical research. Multiple statistical and machine learning methods have been applied for this purpose including consensus clustering [250], linear classifier [251], LASSO regression modeling [252], and deep learning [253]. These methods have been applied to studies on cancers, including breast [250], lung [252], and colorectal [253]. The studies not only demonstrated that integration of morphological features extracted from digitized pathology images and -omics data can improve the accuracy of prognosis but also provided insights on the molecular basis of cancer cell and tissue organizations. For instance, Yuan *et al.* [251] showed that morphological information on TILs combined with gene expression data can significantly improve prognosis prediction for ER-negative breast cancers while the distribution patterns for TILs and the related genomics information are characterized for multiple cancers in [152]. These works led to new directions on integrative genomics for both precision medicine and biological hypothesis generation.

As an extension of the work that is already underway using multi-modal combinations of image and genomic signatures to help support the classification of pathology specimens, there have been renewed efforts to develop reliable, content-based retrieval (CBR) strategies. These strategies aim to automatically search through large reference libraries of pathology samples to identify previously analyzed lesions which exhibit the most similar characteristics to a given query case. They also support systematic comparisons of tumors within and across patient populations while facilitating future selection of appropriate patient cohorts. One of the advantages of CBR systems over traditional classifier-based systems is that they enable investigators to interrogate data while visualizing the most relevant profiles [254]. However, CBR systems have to deal with very large and high-dimensional datasets, the complexity of which can easily render simple feature concatenation inefficient and insufficiently robust. It is often desirable to utilize hashing techniques to encode the high-dimensional feature vectors extracted from computational imaging signatures and genomic profiles so that they can be encapsulated into short binary vectors, respectively. Hashing-based retrieval approaches are gaining popularity in the medical imaging community due to their exceptional efficiency and scalability [255].

## VIII. Concluding Remarks & Future Directions

Medical imaging informatics has been driving clinical research, translation, and practice for over three decades. Advances in associate research branches highlighted in this study promise to revolutionize imaging informatics as known today across the healthcare continuum enabling informed, more accurate diagnosis, timely prognosis, and effective treatment planning. Among AI-based research-driven approaches that have obtained approval from the Food and Drug Administration (FDA), a significant percentage involves medical imaging informatics [256]. FDA is the US official regulator of medical devices and more recently software-as-a-medical-device (SAMD) [257]. These solutions rely on machine- or deep-learning methodologies that perform various image analysis tasks, such as image enhancement (e.g. SubtlePET/MR, IDx-DR), segmentation and detection of abnormalities (e.g. Lung/LiverAI, OsteoDetect, Profound AI), as well as estimation of likelihood of malignancy (e.g. Transpara). Radiology images are mostly addressed in these FDA-approved applications, and, to a lower degree, digital pathology images (e.g. Paige AI). Table III summarizes existing FDA-approved AI-based solutions. We expect significant growth in systems obtaining FDA-approval these numbers in the near future.

Hardware breakthroughs in medical image acquisition facilitate high-throughput and high-resolution images across imaging modalities at unprecedented performance and lower induced radiation. Already deep in the big medical data era, imaging data availability is only expected to grow, complemented by massive amounts of associated data-rich EMR/ EHR, -omics, and physiological data, climbing to orders of magnitude higher than what is available today. As such, the research community is struggling to harness the full potential of the wealth of data that are now available at the individual patient level underpinning precision medicine.

Keeping up with storage, sharing, and processing while preserving privacy and anonymity [258], [259], has pushed boundaries in traditional means of doing research. Driven by the overarching goal of discovering actionable information, afore-described challenges have triggered new paradigms in an effort to standardize involved workflows and processes towards accelerating new knowledge discovery. Such initiatives include multi-institutional collaboration with extended research teams’ formation, open-access datasets encompassing well-annotated (extensible) large-cohorts, and reproducible and explainable research studies with analysis results augmenting existing data.

Imaging researchers are also faced with challenges in data management, indexing, query and analysis of digital pathology data. One of the main challenges is how to manage relatively large-scale, multi-dimensional data sets that will continue to expand over time since it is unreasonable to exhaustively compare the query data with each sample in a high-dimensional database due to practical storage and computational bottlenecks [255]. The second challenge is how to reliably interrogate the characteristics of data originating from multiple modalities.

In that sequence, data analytics approaches have allowed the automatic identification of anatomical areas of interest as well as the description of physiological phenomena, towards in-depth understanding of regional tissue physiology and pathophysiology. Deep learning methods are currently dominating new research endeavours. Undoubtedly, research in deep learning applications and methods is expected to grow, especially in in view of documented advances across the spectrum of healthcare data, including EHR [260], genomic [261], [262], physiological parameters [263], and natural language data processing [264]. Beyond the initial hype, deep learning models managed in a short time to optimize critical issues pertaining to methods generalization, overfitting, complexity, reproducibility and domain dependence.

However, the primary attribute behind deep learning success has been the unprecedented accuracy in classification, segmentation, and image synthesis performance, consistently, across imaging modalities, and for a wide range of applications.

Toward this direction, transfer learning approaches and uptake in popular frameworks supported by a substantial community base has been catalytic. In fact, fine-tuning and feature extraction transfer learning approaches as well as inference using pre-trained networks can be now invoked as would any typical programming function, widening the deep learning research base and hence adoption in new applications.

Yet, challenges remain, calling for breakthroughs ranging from explainable artificial intelligence methods leveraging advanced reasoning and 3D reconstruction and visualization, to exploiting the intersection and merits of traditional (shallow) machine learning techniques performance and deep learning methods accuracy, and most importantly, facilitating clinical translation by overcoming generalization weaknesses induced by different populations. The latter potentially being due to training with small datasets.

At the same time, we should highlight a key difference in the medical domain. Deep learning-based computer vision tasks have been developed on “enormous” data of natural images that go beyond ImageNet (see for example the efforts of Google, and Facebook). This paradigm is rather worrying as in the medical domain matching that size is not readily possible. While in medicine we can still benefit from advances in transfer learning methods and computational efficiency [265], [266] in the future we have to consider how can we devise methods that rely on fewer data to train that can still generalize well. From an infrastructure perspective, computational capabilities of exascale computing driven by ongoing deep learning initiatives, such as the CANDLE initiative, project revolutionary solutions [267].

Emerging radiogenomics paradigms are concerned with developing integrative analytics approaches, in an attempt to facilitate new knowledge harvesting extracted from analysing heterogeneous (non-imaging), multi-level data, jointly with imaging data. In that sequence, new insights with respect to disease aetiology, progression, and treatment efficacy can be generated. Toward this direction, integrative analytics approaches are systematically considered for in-silico modelling applications, where biological processes guiding, for example, a tumour expansion and metastasis, need to be modelled in a precise and computationally efficient manner. For that purpose, investigating the association between imaging and -omics features is of paramount importance towards constructing advanced multi-compartment models that will be able to accurately portray proliferation and diffusion of various cell types’ subpopulations.

In conclusion, medical imaging informatics advances are projected to elevate the quality of care levels witnessed today, once innovative solutions along the lines of selected research endeavors presented in this study are adopted in clinical practice, and thus potentially transforming precision medicine.

## Figures and Tables

**TABLE I:** Summary of Imaging Modalities Characteristics

	Technology	Anatomies	Dimensionality	Cost per Scan*	Storage Requirements
**X-ray**	Produces images by measuring the attenuation of X-rays through the body, via a detector array [9].	Most organs	2D, 2D+t	$15–385	Upto~lGB
**CT**	Creates 2D cross-sectional images of the body by using a rotating X-ray source and detector [25].	Most organs	2D, 3D, 4D	$57–385	Up to 10s of GBs
**Ultrasound**	A transducer array emits acoustic pulses and measures the echoes from tissue scatters [9].	Most Organs	2D, 2D+t, 3D, 4D	$57–230, $633–1483 (with endoscope)	Up to GBs
**MRI**	Use a magnetic field to align protons; RF and gradient pulses are used to selectively excite protons in tissues and blood in order to measure their spatially encoded nuclear magnetic resonance signals	Most organs	3D, 4D	$32–691	Up to 10s of GBs
**Nuclear**	Measures the emission of gamma rays through decay of radioisotopes introduced into the body via external detectors/Gamma cameras [9]	All organs with radioactive tracer uptake	2D, 3D, 4D	$182–1375	Up to GBs
**Microscopy**	Typically uses an illumination source and lenses to magnify specimens before capturing an image [9]	Primarily biopsies and surgical specimens	2D, 3D, 4D	$248–482, $642–1483 (with endoscope)	Can be >1TB

MRI: Magnetic Resonance Imaging, CT: Computer Tomography, RF: Radiofrequency.

* Actual costs vary across providers, countries, and specific imaging parameters. Cost estimates obtained from https://www.medicare.gov/.

**TABLE II:** Selected Deep Learning Methods for Medical Image Segmentation and Classification

Year - [REF] Author	Disease	Imaging Data	Patients	DL Method	Segmentation/Classification	Description
1995 - [100] Lo *et al*	Lung Cancer	X-ray	55	2 layer CNN	Nodules detection in a patch fashion	First ever attempt to use CNN for medical image analysis
2015 - [104] Ronneberger *et al*	Cells	Electron and optical microscopy	30/35	U-net	Segmentation of EM images and cell tracking	Image to image tasks architecture depicting exceptional segmentation performance even with limited data
2016 - [118] Shin *et al*	Interstitial Lung Disease	CT	120 (905 slices)	Transfer learning (AlexNet, GoogleNet, CifarNet CNNs)	Interstitial lung disease binary classification	Showed that networks pre-trained on natural image data could be succesfully used on medical data
2016 - [122] Dou et *al*	Cerebral Microbleeds	MRI	320	Two-stage: 1) 3D Fully-convolutional network (FCN), 2) 3D CNN	3D FCN for candidate microbleed detection	A two-stage system used a 3D FCN to detect candidate microbleeds before a 3D CNN was applied to reduce false positives
2016 - [127] Setio *et al*	Pulmonary Cancer	CT	888 scans, 1186 nodules	Two-stage: 1) Feature- engineered candidate detector, 2) Multi-view 2D CNN for false positive reduction	Candidate pulmonary nodules detection	Significantly reduced false positives using fusion of multiple 2D CNNs at different views around a nodule
2017 - [268] Lekadir *et al*	Cardiovascular (carotid artery)	US	56 cases	Four convolutional and three fully connected layers	Characterization of carotid plaque composition	High correlation (0.90) with plaque composition clinical assessment for the estimation of lipid core, fibrous cap, and calcified tissue areas
2017 – [128] Yu *et al*	Melanoma	Dermoscop ic Images	1250 images	Very deep (38/50/101 layers) fully conv. residual network	Binary melanoma classification	Used a very deep residual network (16 residual blocks) to classify melanoma
2017 - [102] Komnitsas *et al*	TBL LGG/ GBM, Stroke	MRI	61/110/ ISLES-SISS data	11-layers, multi-scale 3D CNN with fully connected CRF	Brain lesion segmentation algorithm	Top-performing segmentation results on TBL brain tumours, and ischemic stroke at BRATS and ISLES 2015 challenges
2017 - [246] Lao *et al*	GBM	MRI	112	Transfer learning	Necrosis, enhancement, and edema tumour subregions	Overall survival prognostic signature for patients with Glioblastoma Multiforme (GBM)
2017 - [247] Oakden- Rayner *et al*	Overall Survival	CT (chest)	48	ConvNet transfer learning (3 convolutional and 1 fully connected layers)	Tissue (muscle, body fat, aorta, vertebral column, epicardial fat, heart, lungs)	Predict patients’ 5-year mortality probability using radiogenomics data (overall survival)
2017 - [241] Zhu *et al*	Breast Cancer	DCE-MRI	270	Transfer learning (GoogleNet, VGGNet, CIFAR)	Breast tumour lesions	Discriminate between Luminal A and other breast cancer subtypes
2018 - [112] Chartsias *et al*	Cardiovascular	MRI	100	Various networks	Segmentation of cardiac anatomy	Limited training data when appropriate autonecoding losses are introduced
2020 – [121] McKinney *et al*	Breast Cancer	X-ray	25,856 & 3,097 cases	Ensemble and transfer learning	Breast cancer classification	Cancer prediction on two large datasets with comparison against human readers
2019 - [170] Hekler *et al*	Melanoma	Whole slide H&E tissue imaging	695	Transfer learning (ResNet50)	Binary melanoma classification	Human level performance in discriminating between nevi and melanoma images

US: Ultrasound; MRI: Magnetic Resonance Imaging; DCE-MRI: Dynamic Contrast Enhancement MRI; CT: Computed Tomography; PET: Positron Emission Tomography; GBM: Glioblastoma; LGG: Lower-Grade Glioma; CNN: Convolutional Neural Networks.

**TABLE III:** AI-Based Medical Imaging Systems With FDA-Approval

Software	Company	Imaging Data	Description
SubtlePET/ SubtleMR	Subtle subtlemedical.com	PET/MRI	Enhancement of PET/MR images
LungAI LiverAI	Arterys www.arterys.com	Lung CT Liver CT, MRI	Segmentation of lesions and nodules
AmCAD-UT	AmCad BioMed www.amcad.com.tw	Thyroid ultrasound	Characterisation and assessment of thyroid tissue
IDx-DR	IDx www.eyediagnosis.co	Retinal	Feedback on image quality, and instructions for patient follow-up or referral
icobrain	Icometrix icometrix.com	Brain MRI, CT	Interpretation of CT and MRI brain images
OsteoDetect	Imagen www.lify.io	Wrist X-ray	Detection of distal radius fracture
All	Zebra Medical Vision www.zebra-med.com	CT, X-ray of various diseases	Detection and quantification of abnormalities
Aidoc Head/Chest/Spine/Abdomen	Aidoc www.aidoc.com	Radiology images	Detection of acute abnormalities across the body
ProFound AI	iCAD www.icadmed.com	2D mammograms	Detection of malignancies and calcifications
Transpara	ScreenPoint Medical screenpoint-medical.com	2D and 3D mammograms	Detection and likelihood of cancer
Accipio	MaxIQAI http://www.maxq.ai/	Head CT	Triaging of intracranial haemorrhage
Paige AI	Paige https://paige.ai/	Digital slides	Diagnosis for digital pathology

US: Ultrasound; MRI: Magnetic Resonance Imaging; CT: Computed Tomography; PET: Positron Emission Tomography.
